# A Manual of General Therapeutics, with Rules for Prescribing, and a Copious Collection of Formulæ

**Published:** 1842-07

**Authors:** 


### Art. XII.

*A Manual of General Therapeutics, with Rules for Prescribing, and a Copious Collection of Formulæ.* By D. Spillan, m.d.—*London,* 1841. 8vo, pp. 458.

We cannot laud the volume before us as one of those performances, masterly at once in the conception and execution, which occasionally surprise and delight us—brightly contrasting with the dull and monotonous mediocrity which precedes and follows them; but with the exception of an occasionally disagreeable poverty of facts, thoughts, and illustrations, we can truly describe Dr. Spillan's work as respectable and useful, though not brilliant or very scientific.

Before proceeding to the examination of the more important and practical portions of the work, we shall briefly refer to one or two statements, which, under the semblance of being philosophical, are, in fact, somewhat crude and shortsighted. At page 2, and elsewhere, Dr. Spillan talks very sneeringly of empiricism. If, on these occasions, he refers (which, however, we do not think he does) to the vulgar empiricism of the regular charlatan, whose treatment is chiefly or wholly that of hazard, and has no foundation in observation and analogy, we agree with his remarks. But he ought not to forget, or to express himself as if he were ignorant, that, in the first instance, the whole art and science of medicine, must have been empirical. When, therefore, the author talks of the "entire process of the treatment," of inflammation, "being decidedly rational," (Advertisement, p. viii.) and asserts that "reason points out the antiphlogistic method of treatment (p. 11) in pneumonia, peritonitis, and all inflammations of organs," he employs the common and non-professional modes of expression of everyday life, not those of science.

We are also surprised at the author's tirades against what he calls the "medicine of symptoms." Can there be, for example, a statement more rashly unqualified than the following? "Though we have taken pains in endeavouring to prove that the most dangerous of all therapeutic methods was the medicine of symptoms, and that all medicinal treatment founded on such miserable indications, tended to the destruction of the patient and the disgrace of the healing art," &c. (p. 20.) In this, as in the former case, we blame Dr. Spillan, for not distinguishing between the symptomatic treatment of the ignorant quack, and "symptomatic medicine," as practised and confided in by the intelligent and educated physician. If his remarks relate to the former, they are stale truisms; if to the latter, they are hasty and unphilosophical dogmas. By what means other than by an observation of symptoms, comprehending, of course, what are called physical signs, can any declension of an organ from its physiological condition and action be indicated or ascertained? Does Dr. Spillan mean to assert that, in any case, the "symptom" is not, if rightly interpreted, or whether rightly interpreted or not, *exactly* expressive of the lesion to which it is due? We maintain it to be so in every case, however anomalous that symptom may seem, or however obscure to *us* may be its connexion with the lesion which causes it.

Let us take, in order to illustrate our meaning, the case referred to by Dr. Spillan himself. A man presents himself with headach from gastric derangement. Were the practitioner to regard the cephalalgia alone,

(and that he should regard this and this alone, Dr. Spillan seems to assume as inevitable,) and to apply leeches, perhaps, to the temples without further measures, such blundering practice would prove, *not* that symptomatic medicine was fallacious, but that the practitioner ill understood the science of symptoms, and grossly neglected to enquire after, and to avail himself of, several signs, *besides* the cephalalgia, which must infallibly have been present in the case referred to. Had he pressed his hand, for example, on the man's epigastrium, it is most likely that tenderness would have been complained of; had he enquired if his headach did not come on at a particular period after eating, was not aggravated by particular sorts or quantities of food; had he examined the patient's tongue, &c. his diagnosis, instead of being erroneous, would have been exact. Yet, be it observed, that the practitioner, all the while, would have been dealing with nothing but symptoms; and his error, if he had fallen into error, would have been owing, not to the absence of "symptoms," or to any fallacy in these, but to his ignorance in confining his attention to one symptom only, and to his neglect of others or his misinterpretation of them. We have only to add that Dr. Spillan's own statement (p. 4) seems directly contradictory of his disparaging opinions elsewhere, in regard to the utility and importance of symptoms: "Thus, then," he observes, "it is not alone the lesions found on the dead body, but those also revealed by the symptoms........which the therapeutist should study, as it is these he is called to treat."

Chapter third opens with the following remark: "All pathologists are now agreed that diagnosis is the most certain of all curative indications," &c. Now diagnosis is not, properly speaking, an indication at all, but implies that observation and comparison of symptoms, on which indication is founded. At p. 34 we find Dr. Spillan remarking: "The changes produced by disease on the appearance of the eyes have rarely any other therapeutic value, save that resulting from their diagnostic importance!" Of a like character, is the remark at page 22: "The yellow colour of the tongue observable in jaundice can have no therapeutic value in itself; the indications are necessarily derived from the diagnosis." True; but is Dr. Spillan prepared to maintain that the appearance of the tongue is absolutely of *no* therapeutic consequence, in contributing *its* share along with other signs, in enabling us to form that *diagnosis?* Surely we have here a very incautious over-statement.

This chapter, which treats of the "Therapeutic Indications derived from Disease," exhibits a view of the indications suggested by the morbid phenomena of organic and animal life, the excretions, and the intellectual faculties. Interspersed through it there are some singular observations. Thus, for example, we are told (p. 22), "The brown or black coating of the tongue is sometimes the result of a concentration of strength;" and at page 30 it is stated that "Percussion of the chest, though so useful in diagnosis, is but of secondary utility in treatment!" At p. 32 we are surprised to find Dr. Spillan stating that "the therapeutic indications derived from the urine are but few and not very important." We are informed, in the same page, that "the yellow and saffron-coloured urine, which generally denotes diseases of the liver, is useful only as a diagnostic sign," as if there were any other way in which it would be so. While hæmaturia is referred to and designated as "but a symptom," no other morbid state or appearance of the urine is referred to, and the presence of albumen, in this excretion, is not even hinted at. At p. 33 we are told that "a restless and inconstant attitude of body . . . . . . . . can only be useful by aiding in the diagnosis;" what other end it could be expected to serve, we must profess ourselves profoundly ignorant.

We may quote the following observations on the causes of diseases as therapeutical indications:

"But little," observes the author, "is known of their mode of action, and there are but few of them really indispensable to the production of diseases; most of them may give rise indifferently to all affections. . . . . . . . . .Let us suppose fifty individuals exposed to the influence of one and the same morbific cause, as, for instance, to the influence of cold; we shall find them all attacked by different diseases. Some will have pneumonia, some pleuritis, some erysipelas, and others rheumatism, which circumstances can only be accounted for by supposing that there exists in each a peculiar predisposition previous to the application of the exciting cause; this predisposition does not constitute a morbid state, as previously to the exposure, they were all in perfect health, and probably without such exposure they would have remained so. Thus these two circumstances are necessary, the one to the other, for the production of the disease; still the predisposition is, without doubt, far the more important of the two." (p. 43.)

We pass over the following interesting subjects, which are treated of in succession, and with varying degrees of copiousness and ability: the causes, nature, seat, course, duration, and periods of diseases, considered as therapeutic indications; the circumstances of professions, habits, temperament, and idiosyncrasy and habits, which modify curative indications. We then come to chapter five, the subject of which is the modus operandi of medicines, and here there are some interesting matters presented to us.

Dr. Spillan points out that medicinal actions are of two sorts; the one confined to the point of application, the other extending to remote organs. The circumstance of medicines producing merely local effects is due to the medicinal substance possessing no peculiar activity, or to its being applied in a small dose or to an important organ. With these exceptions, the effect of medicinal substances is to influence remote organs along with, or independently of, the parts where they are primarily applied; and the question is by what mechanism these distant phenomena are effected. By some it is supposed that the nerves, by a mutual *consensus*, either sympathetic or antagonistic, transfer the local impression from one part to another. Another proposed solution of the phenomena is that of mere proximity; as when substances applied to the abdominal parietes, affect the stomach or bladder. The objections to this theory of medicinal actions are stated to be, first, that the circumstance of several substances as iodine, arsenic, mercury being detectible in the blood, is opposed to the idea that these act only at the point of application, since, circulating in the blood, they must come into contact with all parts of the body. Secondly, that after both external and internal exhibition, several medicinal substances appear in the excretions; another proof that, dissolved in the blood, they must have previously perambulated the body, and consequently have operated at many other points than the one of primary application. These facts suffice to prove that when a medicine acts "dynamically," its remote effects cannot be accounted for by nervous sympathy or "nervous conduction." (p. 76.) We should rather say that these facts simply prove that there are *other* modes of explaining remote effects than that of "nervous conduction." According to the existing views in physiology there is but one other way of accounting for the phenomena of remote actions, and this is by supposing that the medicinal substances themselves reach distant organs; and how can this happen unless by these substances being absorbed into the circulation, and thus distributed? And that this is the real method of diffusion is proved by the following facts: 1. By the medicinal substances being found in the blood. 2. By their being found in the secretions and excretions. 3. By their being found deposited in the solid parts of the body. 4. By the effects on remote organs taking place at a perceptibly later period than the local effects. (Dr. Spillan assigns this as one proof; but we do not see that the same argument will not equally apply to the theory of "nervous conduction.") 5. By the circumstance that while neither structural nor functional changes occur at the point of application of the medicinal substance, considerable effects take place in remote parts or organs. Dr. Spillan gives several other facts proving that it is by diffusion in the blood that medicinal substances produce their remote effects. We shall refer to only one or two of these. When a substance is introduced into a part, the circulation of which is allowed to continue, but its nervous connexions are cut off, the systemic phenomena are not the less rapidly and completely manifested; while, in the contrary case, namely that of the blood-vessels being tied, and the nerves left untouched, no systemic effects follow the introduction or application of a medicinal substance. Lastly; medicinal substances introduced directly into the blood, act in just the same way as when taken internally, or exhibited in any other way.

The following are the circumstances which distinguish medicinal actions produced through the circulation and those produced by nervous conduction: 1. When the remote action of a medicinal substance is a sympathetic one, it corresponds, in strength, to a greater or less extent, with the local action. 2. The remote action, in the case of nervous conduction, is so much the more intense, in proportion as the organ which is locally affected by the medicine is important. We omit the third proof referred to by Dr. Spillan, namely, the negative one obtained from section of the nerves, between the point at which the medicinal substance is applied and that at which the remote action takes place; and we give the fourth, and last, in the author's own words: "The remote effects occasioned by a consensus of the nerves, and which are solely the consequence of the local medicinal action, follow always immediately after the local action, cannot take place without this, are not prevented by an artificial plethora," &c. (p. 89.)

At pp. 95 et seq., the question is discussed, "How the blood and medicinal substances react on each other when they meet;" and under this head some interesting facts and views are brought forward. The following is a summary of Dr. Spillan's conclusions: Whenever a medicinal substance, howsoever administered, is found in the secretions, excretions, or solids, retaining all its original properties, we must conclude that it has been merely mixed with the blood. But though such substances suffer no change from the blood, the converse of the proposition does not follow that the blood suffers no change from the presence of the medicinal substance. Thus neutral salts, though undergoing no change themselves, prevent or retard the coagulability of the blood, according to Dr. Stevens's experiments, to which property these salts are supposed to owe their antiphlogistic power; "although," justly adds Dr. Spillan, "for producing such effects they owe something to their property of promoting the secretions." (p. 96.)

Bitters also produce considerable changes on the blood, but the effect is not, the author thinks, so much due to their operation on the constituents of the blood as to their improving the action of the digestive organs. Acids, particularly the vegetable, are always found at least in the urine, combined with bases; and from the fact of mercury never being detectible in the blood, unless the blood itself be entirely decomposed, we must infer that the metal combines intimately with some constituent of the blood, probably with the albumen.

The author then proceeds to the consideration of another very interesting question, the terms of which we shall allow him to state in his own words:

"If we suppose a medicinal substance circulating in the blood, the first question which forces itself on us is, how does this act on the parietes of the vessels? Considering that medicinal action can take place only where there are nerves, we must conclude, *a priori*, that in the larger trunks, and even in the smaller vessels, no medicinal actions go on, there being no nerves on the inner membrane of these vessels. Medicinal substances which enter the circulation can, therefore, only act there, where all change of substance and all reciprocal action between the solids and fluids take place, namely in the capillary vascular system. But it is only the central parts and the peripheric terminations of the nervous system that are in the most intimate and multiplied contact with the capillary system. It is here that the medicinal substances, circulating with other constituents of the blood, enter from these vessels, and, as it were, irrigate the islets of the solid parts lying between them; and through this contact with the nervous terminations, and, one might say, with the interior of the central organs of the nervous system, medicinal action is called forth. Numerous experiments prove that the blood-vessels have no capability of reaction on the application of medicinal substances, one of the factors necessary for medicinal action being wanting, we mean the nerve. The experiments of Fontana, Wedemeyer, Christison, and Macartney establish the same fact." (pp. 98-9.)

Dr. Spillan concludes that medicinal substances produce their actions "through contact with the centres of the nervous system, and its terminations alone." (p. 99.) But he proposes the question whether "all medicines which act on the periphery of the organism, when they come into contact with the nervous endings, will do the same thing when they meet with them, when circulating in the blood;" and thinks, "there are facts which show that medicinal substances taken into the blood excite medicinal actions, in contact with the nervous terminations." (p. 99.) Thus on Dupuytren injecting milk into the veins of a dog, he saw the same movements produced as if it had been applied to his tongue. A strong-scented fluid, injected in like manner, caused the animal to dilate his nostrils and "snuff" around him, in quest of the source of the odour. Hale, on introducing castor oil into a vein of his arm, had shortly after the taste of it in his mouth.

That, in other cases, certain substances act chiefly or solely on the nervous centres, is shown by the fact that opium, introduced into the carotid, kills much sooner than when injected into any other vessel; and this peculiarity Orfila states as characterizing all narcotics.

Still, one of the most singular, at once, and most important properties of several medicinal substances remains unexplained; that, namely, of their operating on particular organs only. The following facts are considered by Dr. Spillan to prove "that particular organs possess a specific susceptibility for certain medicinal substances." 1. Particular substances occasion an increased afflux of blood to the organs on which they chiefly exert their influence, and even excite in them structural changes. 2. Medicines which act on particular organs are occasionally deposited in them. 3. Medicines which promote certain secretions are excreted with these. This last phenomenon, observes Dr. Spillan, "can only be accounted for by the admission, that the nerves of certain organs stand in a peculiar relation to certain medicinal substances." (p. 105.) The truth of this proposition is, according to the author, proved by Höhler's experiments in respect to diuretic medicines, as the vegetable acids, the diuretic salts, squills, garlic, oil of turpentine, &c. The most important conclusions deduced by the author from these facts are:

"The secretions already formed in the blood are more especially attracted by certain organs. Among the secretions which possess peculiar constituents may be reckoned the urine. In the healthy state, urea and uric acid are found in it. Now with respect to the urea, it has been shown by Prevost and Dumas that it exists already formed in the blood, and that it is not in the kidneys merely that it is formed. With respect to this substance, it must now be admitted that it is eliminated by the kidneys, solely for this reason, that between both of them, there exists a species of elective attraction." (pp. 107-8.)

The particular impressibility of certain organs by certain medicinal substances, Dr. Spillan thus endeavours to account for:

"Light and colour are the natural stimuli of the organ of vision, and no other sense is affected by them; sound stimulates the ear and not the eye; sugar makes no impression on the organ of smell, and he who possesses not this latter sense may bathe in rose oil, without experiencing the least odour. If, then, we can understand that a particle of an odour does not affect the eye, and that the organ of smell is insensible to light, we may easily conceive how it happens that a medicinal substance circulating in the blood, though it come in contact with all the organs, affects only some particular organ." (pp. 108-9.)

Chapter vi. is devoted to a consideration of the surfaces adapted for the application of medicinal substances, and to the particulars which naturally grow out of this subject. It is stated (p. 111), that the surfaces proper for the reception of medicinal substances are ten in number, namely, 1, the stomach and intestines; 2, the large intestines alone; 3, the skin; 4, the surface of the eyes; 5, the pituitary membrane; 6, the interior of the mouth; 7, the surface of the air-tubes; 8, the meatus auditorius; 9, the interior of the urethra and bladder; 10, in the female, the vagina and sometimes the cavity of the uterus. These surfaces might have been more succinctly described as comprising or comprised in the mucous and dermoid membranes. The author thinks that the part which the "system of innervation" performs in the operation of medicine has never been sufficiently appreciated.

"When a medicinal substance traverses the interior of the primæ viæ, it is less on the organic fibres which constitute the tissues of the stomach and intestines that it acts, than on the nervous expansions with which it comes in contact. 1. In the stomach, the divisions of the pneumo-gastric nerve transmit its action to the medulla oblongata: this action may produce some change in the state of the latter, and impart to it a new power and an unusual force of innervation. 2. On the intestinal surface the medicine will come in contact with nerves communicating with the spinal cord. The modifications produced in these nerves will soon become common to the spinal cord, which will take on another mode of action, and give rise to perceptible changes over all the parts of the animal system. 3. Every medicine on coming into the stomach touches the irradiations of the solar plexus, which will of course undergo some modification, and accordingly its influence will assume an altered character. The impression so made on the solar plexus extends to all the plexuses of the ganglionic nerves. The entire system of innervation, in consequence of the impressions of a medicine, thus acquires an accidental or new activity, which is diffused over the entire animal economy. Thus we see the importance which should be attached to the operation of medicinal substances on the nervous plexuses, the spinal marrow, and the medulla oblongata. In conclusion, it may be well to remark, that for the application of certain medicines, there is a vast difference between a surface which corresponds with the medulla oblongata and a surface which has its communications confined to the spinal cord; thus tartar emetic in the dose of one or two grains usually produces vomiting, when given by the mouth: whilst, if given in the form of lavement, in the dose of six, eight, or ten grains, it no longer vomits; or if it do, it is not till six or more hours after the introduction of the substance by the anus, and the effect is then produced by absorption. The reason is this: in the stomach it comes in contact with the divisions of the pneumo-gastric nerve, which transmit the impression to the medulla oblongata; such connexion does not exist for the intestinal surface." (pp. 112-3.)

In chapters vii. and viii. the "effects of medicinal substances" and the "therapeutic action of medicines" are respectively treated of. Both chapters contain some unnecessary refinements and obscurities, and some trite truths. In chapter vii. the effects of medicines are distinguished into primary and secondary; and, so far as we can perceive, the secondary or curative effects of this chapter are identical with the therapeutic ones of the succeeding. At p. 136 the author remarks: "the immediate or primary effects of a medicine are sure to take place whenever such medicine is employed; if any difference should occur, it will be in degree not in kind. Thus an excitant will always stimulate tissues, purgatives will always irritate the intestinal surface. The curative effects are not thus constant and uniform." But, in fact, drugs have in one sense no other than primary effects, whether these take place in the stomach or only after reaching the nervous centres, by the medium of the circulation; for the secondary or curative effect is, in truth, just the physiological one. In other words, it is not the medicinal substance that accomplishes a cure directly; but a cure follows in consequence of certain physiological actions which the medicinal substance has been instrumental in exciting or modifying in the living organs. There is no mystery in this, so far at least as regards the mere relation in which the drug and the vital organ stand to each other.

